# A quantitative and efficient approach to select MIRU–VNTR loci based on accumulation of the percentage differences of strains for discriminating divergent *Mycobacterium tuberculosis* sublineages

**DOI:** 10.1038/emi.2017.58

**Published:** 2017-07-26

**Authors:** Xin-Ling Pan, Chun-Lei Zhang, Chie Nakajima, Jin Fu, Chang-Xia Shao, Li-Na Zhao, Jia-Yi Cui, Na Jiao, Chang-Long Fan, Yasuhiko Suzuki, Toshio Hattori, Di Li, Hong Ling

**Affiliations:** 1 Department of Microbiology, Wu Lien-Teh Institute, Harbin Medical University, Heilongjiang Provincial Key Laboratory of Infection and Immunity, Key Laboratory of Pathogen Biology, Harbin 150081, China; 2 Department of Clinical Laboratory, Harbin Chest Hospital, Harbin 150081, China; 3 Division of Bioresources, Hokkaido University Research Center for Zoonosis Control, Sapporo 0010020, Japan; 4 The Global Station for Zoonosis Control, Hokkaido University Global Institution for Collaborative Research and Education, Sapporo 0600808, Japan; 5 Department of Neurology, The Second Affiliated Hospital of Harbin Medical University, Harbin 150026, China; 6 Graduate School of Health Science Studies, Kibi International University, Takahashi 7168508, Japan

**Keywords:** Beijing genotype, *Mycobacterium tuberculosis*, mycobacterial interspersed repetitive units (MIRU)–variable number tandem repeat (VNTR), sublineage

## Abstract

Although several optimal mycobacterial interspersed repetitive units–variable number tandem repeat (MIRU–VNTR) loci have been suggested for genotyping homogenous *Mycobacterium tuberculosis,* including the Beijing genotype, a more efficient and convenient selection strategy for identifying optimal VNTR loci is needed. Here 281 *M. tuberculosis* isolates were analyzed. Beijing genotype and non-Beijing genotypes were identified, as well as Beijing sublineages, according to single nucleotide polymorphisms. A total of 22 MIRU–VNTR loci were used for genotyping. To efficiently select optimal MIRU–VNTR loci, we established accumulations of percentage differences (APDs) between the strains among the different genotypes. In addition, we constructed a minimum spanning tree for clustering analysis of the VNTR profiles. Our findings showed that eight MIRU–VNTR loci displayed disparities in *h* values of ≥0.2 between the Beijing genotype and non-Beijing genotype isolates. To efficiently discriminate Beijing and non-Beijing genotypes, an optimal VNTR set was established by adding loci with APDs ranging from 87.2% to 58.8%, resulting in the construction of a nine-locus set. We also found that QUB11a is a powerful locus for separating ST10s (including ST10, STF and STCH1) and ST22s (including ST22 and ST8) strains, whereas a combination of QUB11a, QUB4156, QUB18, Mtub21 and QUB26 could efficiently discriminate Beijing sublineages. Our findings suggested that two nine-locus sets were not only efficient for distinguishing the Beijing genotype from non-Beijing genotype strains, but were also suitable for sublineage genotyping with different discriminatory powers. These results indicate that APD represents a quantitative and efficient approach for selecting MIRU–VNTR loci to discriminate between divergent *M. tuberculosis* sublineages.

## INTRODUCTION

The extensive spread of multidrug-resistant tuberculosis in Asia is predominantly driven by the rapid spread of the *Mycobacterium tuberculosis* Beijing genotype.^1, 2, 3, 4^ Beijing genotype strains have been proposed to have an increased capacity to acquire drug resistance, have an increased transmission ability, exhibit hypervirulence and result in a more rapid progression to disease after infection.^5, 6, 7, 8, 9, 10^ However, the association of infection with the Beijing strain with drug resistance and specific pathobiological or epidemiological characteristics has not been systematic.^11^ Although this genotype is associated with drug resistance,^12, 13, 14, 15, 16^ relatively weak associations have been reported in other geographic settings.^17, 18, 19^ The heterogeneity suggests the existence of substantial intra-lineage biogeographical diversity affecting pathobiological properties.^20^ The Beijing genotype includes several divergent sublineages,^21, 22, 23^ the phenotypic differences among which have not been characterized. To date, it remains unclear whether the distribution of sublineages in different geographic areas is causally related to the variations in tuberculosis (TB) transmission dynamics and/or the prevalence of drug-resistant TB.

To explore the mechanisms associated with the spread of *Mycobacterium* spp. and to clarify the correlation between genotypes and phenotypes, extensive genotyping studies have been performed.^6, 20, 24, 25^ In China, the prevalence of the Beijing genotype varies from 25% to 92% among geographic areas,^19^ with the distribution of the Beijing sublineages also varying geographically.^20, 26, 27^ This could lead to misunderstandings of the phenotypic features of *M. tuberculosis* strains and the Beijing genotype epidemic.

Over the previous decade, the molecular epidemiology of *M. tuberculosis* advanced markedly,^28, 29^ mainly because of the development of variable number tandem repeat (VNTR) typing and its application to mycobacterial interspersed repetitive units (MIRUs).^30^ Compared with IS6110-RFLP (insertion sequence 6110-restriction fragment length polymorphism) and spoligotyping, MIRU–VNTRs display a higher discriminatory power, especially in strains with low copy numbers of IS6110, and are suitable for long-term epidemiologic analyses because of their slightly slower evolution.^19, 28, 31, 32^ VNTR genotyping is based on the discrimination power of a locus in a certain geographic region where a certain type of *M. tuberculosis* is dominant. However, there are geographic polymorphisms in certain VNTR loci, and even the standard 15- and 24-locus VNTR sets might not be optimal for discriminating *M. tuberculosis* isolates in geographic areas where the Beijing genotype strains are highly endemic.^33, 34, 35, 36^ In addition, choosing a locus based only on its discriminatory power might result in genotyping errors for Beijing and non-Beijing family strains because of the different genetic features of the populations.

Single nucleotide polymorphisms (SNPs) have been used for genotyping sublineages of the Beijing family.^37, 38, 39, 40^ An advantage of SNPs is that they allow the unambiguous assignment of a particular clone to a certain lineage; however, SNP typing is less informative in molecular epidemiology studies because of its low discriminatory power.^41^

Luo *et al.*^42^ conducted genotyping of Beijing strains by combining SNPs and VNTR, showing that SNPs in modern Beijing strains correctly classified clusters that were previously misclassified because of the limited discriminatory power and/or homoplasy of VNTR.^42^ Therefore, the combinatorial use of VNTR and SNPs might allow discrimination of Beijing and non-Beijing strains, as well as sublineages of Beijing genotypes.

Previously, we defined a 15-locus VNTR set to effectively distinguish *M. tuberculosis* isolates in Heilongjiang Province,^19^ finding that Beijing family strains account for 90% of *M. tuberculosis*. In addition, there is a heavy burden of drug-resistant TB in Heilongjiang Province.^43^ Accordingly, we focused on exploring genotype markers to distinguish Beijing and non-Beijing family isolates and developing appropriate genotyping approaches for areas with a high burden of Beijing genotype lineages. It is important to understand the possible mechanisms by which *M. tuberculosis* and drug-resistant TB spread; therefore, in this study, we established a new approach to evaluate the power of a VNTR locus and SNP genotyping for discriminating Beijing and non-Beijing family strains, as well as Beijing sublineages.

## MATERIALS AND METHODS

### Collection of *M. tuberculosis* clinical isolates and identification of the Beijing genotype and sublineages

This study included 281 *M. tuberculosis* samples isolated between May 2007 and October 2009 from 281 patients from various regions of Heilongjiang Province who had been diagnosed with pulmonary TB in the Harbin Chest Hospital.^27^ Of these strains, 200 were described in our previous work,^19^ with an additional 81 strains described here. The Beijing family strains were determined by RD105 deletion and the Rv0679c SNP.^27, 44^ Non-Beijing family strains were identified by a long sequence polymorphism as described.^45^ Sublineages of the Beijing family strains (sequence types (STs)) were previously determined by detection of nine SNPs that are only present in Beijing family strains.^27, 38^ All primers used for polymerase chain reactions for determination of genotypes and sublineages are shown in Supplementary Table 1.

### MIRU–VNTR typing

In this study, we used 22 MIRU–VNTR loci (Table 1) for the genotyping of the 81 isolates. The 200 isolates previously genotyped by 15 VNTR loci were analyzed with seven new VNTR loci (VNTR3820, QUB3232, VNTR4120, QUB11a, QUB18, QUB4156 and Mtub24), resulting in the availability of 281 isolates with 22 VNTR loci for analysis.^19^ MIRU–VNTR allelic diversity (*h*) at a given locus was calculated as follows: *h*=1−∑*x*_*i*_^2^ [*n*/(*n−*1)], where *x*_*i*_ is the frequency of the *i*th allele at the locus, and *n* is the number of isolates.^46^ Discrimination of the locus combination was calculated using the Hunter–Gaston discriminatory index (HGDI):^46^


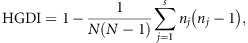


where *N* is the total number of isolates in the typing method, *s* is the number of distinct patterns discriminated by MIRU–VNTR and *n*_*j*_ is the number of isolates belonging to the *j*th pattern.

Accumulation of the percentage differences (APDs) of the strains with the same repeat-number pattern at a certain VNTR locus between category A and category B were obtained as follows:


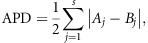


where *s* is the total number of distinct repeat patterns, *A*_*j*_ (*B*_*j*_) is the percentage of *A (B)* category strains with the *j*th repeat pattern from all the strains of the *A (B)* category.

### Minimum spanning tree (MST) construction and analysis of locus-set(s) discrimination

For classification of the strains of different genotypes based on a VNTR locus set, a minimum spanning tree (MST) was created using Bionumerics software version 6.6 (Applied Maths, St. Martens-Latem, Belgium) for the 281 *M. tuberculosis* strains based on VNTR genotyping and ST typing.^47^ The rules for tree construction were the same as those previously reported.^47^ An MST connects all the genetic profiles, such that the global genetic distance between all the branches is minimized.

## RESULTS

### Discriminatory power of the 22 VNTR loci for Beijing and non-Beijing isolates

A total of 281 *M. tuberculosis* isolates were analyzed, with 252 strains belonging to the Beijing genotype strains and 29 strains identified as the Euro-American genotype. We previously examined the discriminatory power of 15 VNTR loci and defined a combination of loci for genotyping *M. tuberculosis* isolates in Heilongjiang Province.^19^ Here we examined additional VNTR loci to identify a more suitable set that exhibited better discriminatory power for genotyping local isolates. We first evaluated the discriminatory power of seven previously unused loci to investigate the local isolates, followed by re-evaluation of the 22 loci (15 previously evaluated and seven new) using a larger sample population (Table 1). Among the seven loci, three (VNTR3820, QUB3232 and VNTR4120) displayed an extremely high degree of discriminatory power for the Beijing and non-Beijing isolates in the study area. QUB11a and QUB18 showed moderate discrimination (0.3<*h*<0.6), whereas the other two loci, QUB4156 and Mtub24, showed low discrimination (*h*≤0.3).

We also found that some loci exhibited highly variable discrimination between Beijing and non-Beijing isolates. For example, eight loci displayed *h*-value disparities of ≥0.2 between the two isolate types. In addition, the loci QUB18, Mtub21 and MIRU40 displayed moderate or low discrimination values for Beijing isolates, but high discrimination values for non-Beijing isolates. Three VNTR loci (VNTR3820, QUB3232 and VNTR4120) were excluded from further analyses to avoid the influence of high allelic diversity on the genotyping results.

### VNTR loci for discriminating Beijing and non-Beijing isolates

Because the VNTR loci exhibited different discriminatory powers between Beijing and non-Beijing isolates, we attempted to determine the optimal VNTR loci for this process. Given that there are differences in allelic diversity (*h*) and repeat number at some VNTR loci between Beijing and non-Beijing isolates (Supplementary Figure S1), we hypothesized that the difference, specifically the accumulative difference in allelic diversity, represents the discriminative capacity of the locus for various isolate types. Therefore, we calculated the APD for each locus as described in the ‘Materials and Methods’ section.

**Figure 1:**
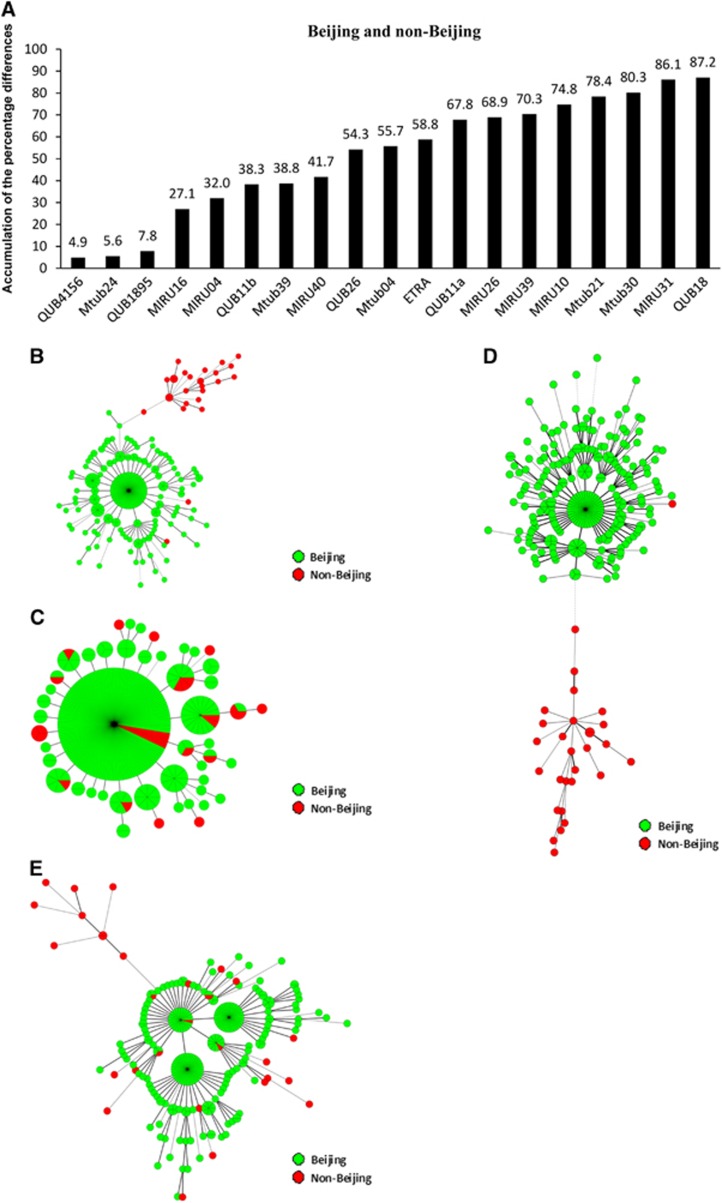
VNTR loci for discriminating the Beijing and non-Beijing family strains. The accumulations of the percentage differences of 19 VNTR loci between the Beijing and non-Beijing strains (**A**). MSTs of the 281 strains based on QUB18, MIRU31, Mtub30, MIRU10, MIRU39 and Mtub21 (**B**); QUB4156, Mtub24, QUB1895, MIRU16 and MIRU04 (**C**); QUB18, MIRU31, Mtub30, Mtub21, MIRU10, MIRU39, MIRU26, QUB11a and ETRA (**D**); and QUB4156, Mtub24, QUB1895, MIRU16, MIRU04, QUB11b, Mtub39 and MIRU40 (**E**). Beijing and non-Beijing strains are indicated in different colors.

To confirm our hypothesis, we constructed an MST using all *M. tuberculosis* isolates. We found that the Beijing and non-Beijing isolates were separated into two main populations when we used QUB18, MIRU31, Mtub30, MIRU10, MIRU39 and Mtub21, with APD values >70% (Figure 1A). MST analysis indicated that 93.1% (27/29) of non-Beijing isolates belonged to one branch, whereas 100% (252/252) of Beijing isolates belonged to another branch (Figure 1B). The APDs of the locus set, including the other five loci (QUB4156, Mtub24, QUB1895, MIRU16 and MIRU04), were low (4.9%–32.0%). These results indicated that the set exhibited a far worse capacity for discrimination relative to that of the former set (Figure 1C).

Similar results were obtained using APD values for seven-, eight-, and nine-locus sets. For example, we chose nine loci based on the descending order of the APD values. The APDs, arranged from high to low for the addition of each locus (that is, QUB18, MIRU31, Mtub30, Mtub21, MIRU10, MIRU39, MIRU26, QUB11a and ETRA), ranged from 87.2% to 58.8%. In addition, MST analysis indicated the same two branches, with 96.6% of the non-Beijing isolates found on one branch (Figure 1D). However, when we chose loci based on the APD values in ascending order, the MST for the loci (QUB4156, Mtbu24, QUB1895, MIRU16, MIRU04, QUB11b, Mtub39 and MIRU40) with APDs ranging from 4.9% to 41.7% did not indicate clusters of Beijing or non-Beijing isolates and failed to classify isolates of each genotype to specific MST branches (Figure 1E). We ultimately determined that the VNTR loci with APDs >56% could efficiently discriminate the Beijing and non-Beijing isolates.

### VNTR loci for deep discrimination of Beijing sublineages

We previously found that Beijing genotype isolates account for ~90% of all *M. tuberculosis* clinical isolates and that ST10 was dominant (63.2%) among the 10 STs found in the study area.^27^ Here we combined ST and VNTR genotyping to precisely classify clinical isolates and identify appropriate VNTR loci for areas where Beijing genotype isolates are dominant.

**Figure 2:**
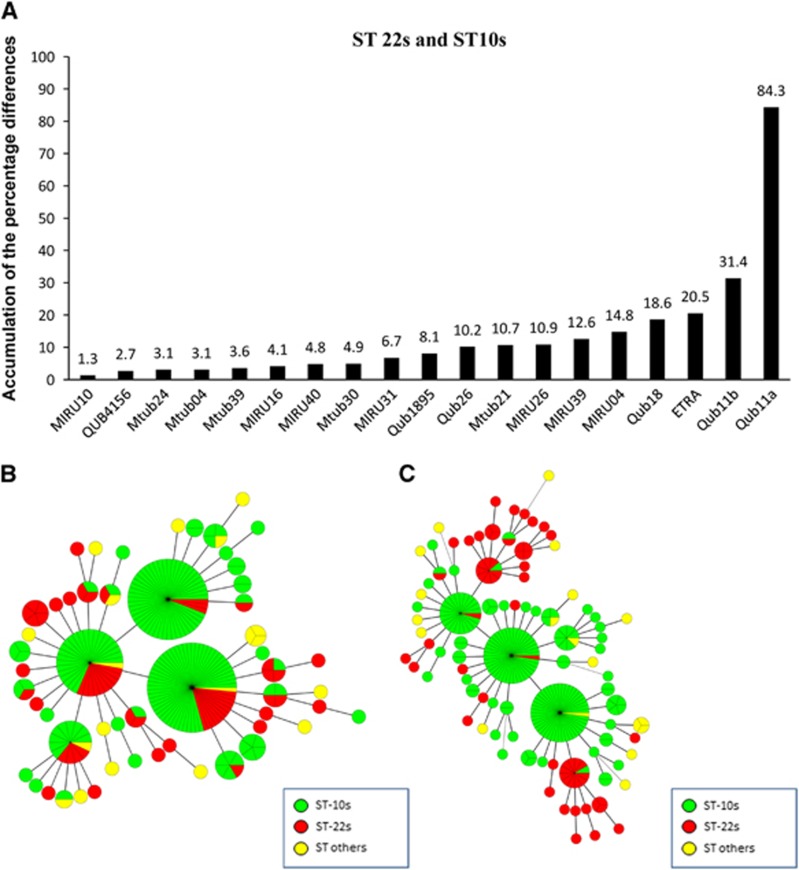
VNTR loci for discriminating between the ST10s and ST22s strains of the Beijing family. The accumulation of the percentage differences of the 19 VNTR loci between the ST10s and ST22s strains (**A**). MSTs of the 252 Beijing strains based on QUB11b, ETRA and QUB18 (**B**); QUB11a, QUB11b, ETRA and QUB18 (**C**). ST22s, ST10s and ST others strains are indicated in different colors.

**Figure 3:**
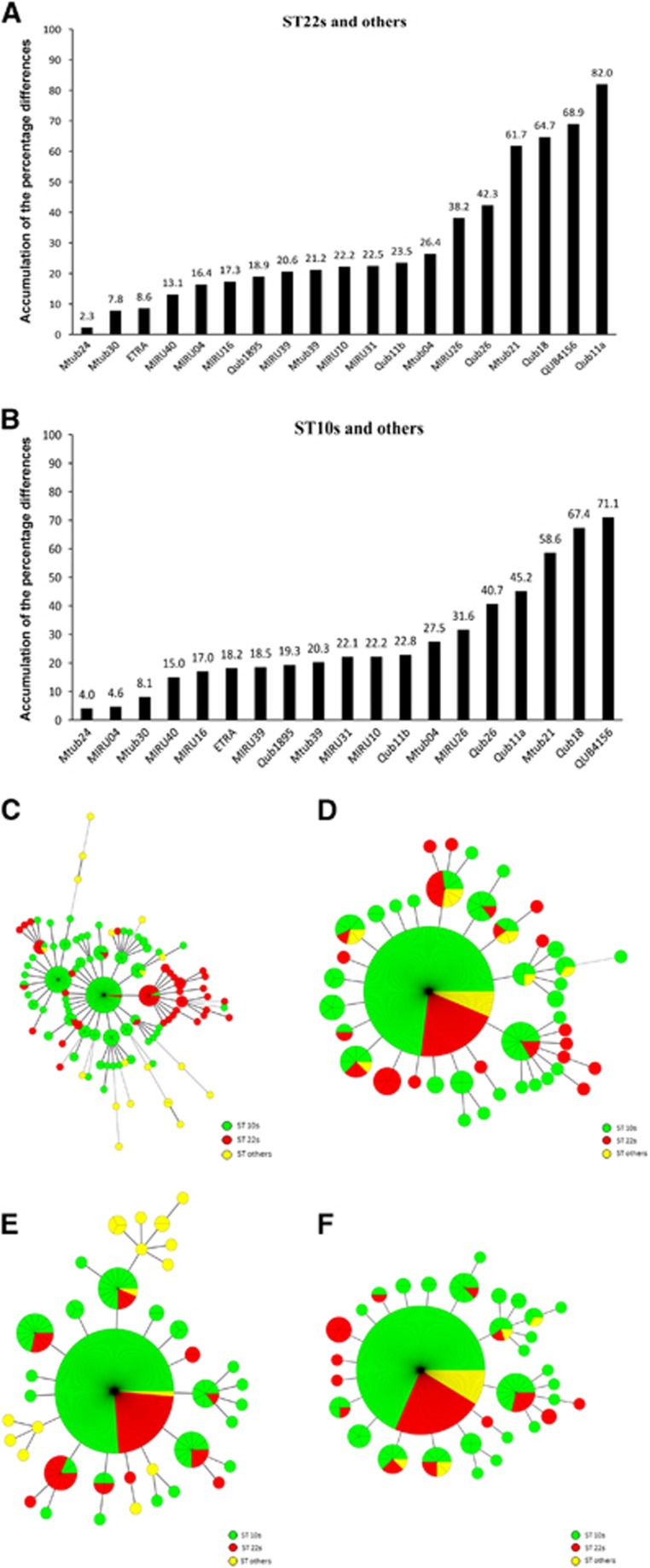
VNTR loci for discriminating ST22s or ST10s strains from ST others strains of the Beijing family. The accumulation of the percentage differences of 19 VNTR loci between ST10s and ST others strains (**A**) and between ST22s and ST others strains (**B**). MSTs of the 252 Beijing strains based on QUB11a, QUB4156, QUB18, Mtub21 and QUB26 (**C**); Mtub24, ETRA, Mtub30, MIRU40 and MIRU04 (**D**); QUB4156, QUB18 and Mtub21 (**E**); and Mtub24, MIRU04, Mtub30 and MIRU40 (**F**). ST22s, ST10s, and ST others strains are indicated in different colors.

We first calculated the frequencies of strains with a certain repeat number at a particular VNTR locus to classify the ST strains (Supplementary Figure S2). All the Beijing isolates were grouped into three ST categories: ST10s (173 isolates, including ST10, STF and STCH1), ST22s (61 isolates, including ST22 and ST8) and ST others (18 isolates, including ST25, ST26, ST11, ST19 and ST3) (Supplementary Table S2). We then compared the pairwise difference in APDs of the VNTR loci between ST categories (ST10s vs ST22s, ST22s vs ST others and ST10s vs ST others) (Figures 2A,3A and 3B). We found that QUB11a was the most powerful locus, with an APD of 84.3% and high level of discrimination between ST10s and ST22s strains (Figure 2A). The APDs for the other VNTR loci were from 1.3% to 31.4% and exhibited lower levels of discrimination for different ST categories.

**Figure 4:**
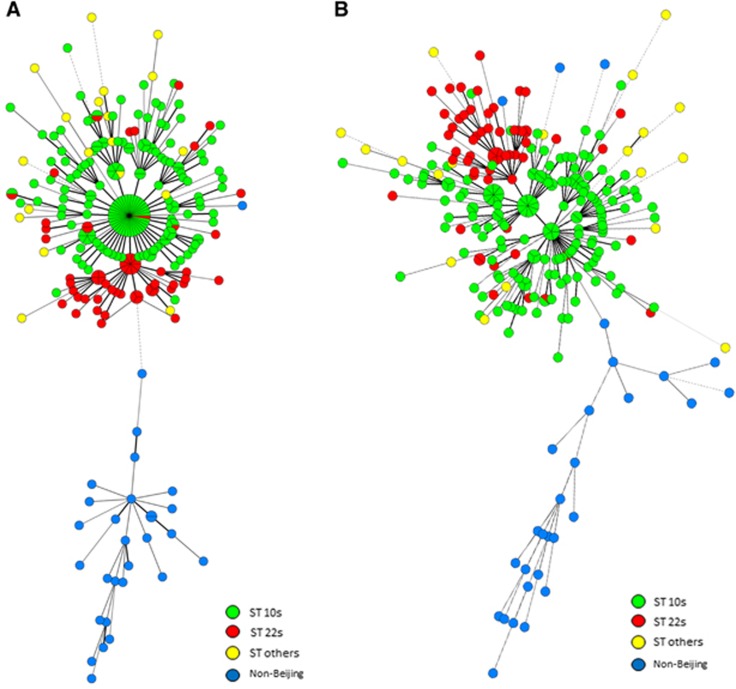
MSTs of the 281 strains based on QUB11a, QUB18, Mtub21, MIRU26, MIRU31, MIRU39, ETRA, MIRU10 and Mtub30 (**A**); and QUB11a, QUB18, QUB4156, Mtub21, QUB26, MIRU26, QUB11b, MIRU31 and Mtub04 (**B**). Non-Beijing family and sublineages of the Beijing family strains are indicated in different colors.

To confirm the power of QUB11a and the methodology for locus selection, we chose additional loci based on APDs in descending order. For example, QUB11b, ETRA and QUB18 showed APDs in the range of 31.4% to 18.6%. The MST did not indicate clustering of the strains from the same sublineages and failed to assign strains of each sublineage to a specific MST branch (Figure 2B). Using QUB11a, we observed that ST10s and ST22s strains formed distinct groups, with ST10s strains divided into three clusters and ST22s strains belonging to two separate clusters (Figure 2C). Using the same strategy, we found five loci capable of discriminating ST22s and ST others within the study area (that is, QUB11a, QUB4156, QUB18, Mtub21 and QUB26; APDs: 82.0%–42.3%) (Figures 3A and 3C). By contrast, Mtub24, ETRA, Mtub30, MIRU40 and MIRU04 were unable to discriminate these two groups (Figure 4D). These findings indicated that loci with APDs >40% efficiently discriminated ST22s strains from ST others strains.

The APD order for loci capable of discriminating ST10s strains from ST others strains was similar with that of ST22s strains, except for QUB11a (Figures 3A and 3B). We found three loci capable of discriminating ST10s from ST others (that is, QUB4156, QUB18 and Mtub21; APDs: 71.1%–58.6%) (Figure 3E). As expected, the four-locus set (Mtub24, MIRU04, Mtub30 and MIRU40) exhibiting low APDs did not distinguish ST10s strains from ST others strains (Figure 3F).

We concluded that VNTR loci used to discriminate dominant Beijing genotype sublineages in the study area should have APDs >40%. QUB11a could discriminate ST22s strains from ST10s strains, whereas three loci (QUB4156, QUB18 and Mtub21) were able to discriminate ST others strains from ST10s or ST22s strains. Furthermore, addition of QUB26 did not influence the discrimination of Beijing genotype sublineages (data not shown) but did increase the discriminatory power. Therefore, a combination of QUB11a, QUB4156, QUB18, Mtub21 and QUB26 was used as the first-line set for genotyping the Beijing sublineages.

### Genotyping loci for discriminating locally endemic sublineages

According to the locus selection strategy described here and the loci exhibiting discriminatory power for local sublineages, we found that the nine-locus combination with the top APDs between Beijing and non-Beijing genotype strains classified all the strains into two clusters, with 100% of the Beijing strains belonging to one branch and 96.6% of the non-Beijing strains belonging to another branch (HGDI: 0.979; cluster rate: 30%) (Figure 4A), and that the ST22s and ST10s strains were effectively divided. Another nine-locus combination with the highest APDs among the sublineages of Beijing genotype strains also discriminated Beijing from non-Beijing strains (Figure 4B). In this analysis, 89.7% of the non-Beijing strains were found on one branch and the ST22s strains accounted for 88% of the ST22s branch (HGDI and cluster rate: 0.997 and 14.5%, respectively).

## DISCUSSION

MIRU–VNTR genotyping using the standardized 24-locus set has become an international standard and is currently applied for *M. tuberculosis* genotyping in China and worldwide.^48^ Both 12-locus and 15-locus sets have been proposed as alternatives for rapid and high-throughput genotyping,^49^ with a recent study indicating that a 15-locus VNTR set could be very useful for the genotyping of *M. tuberculosis* complex strains in China and that a new 19-locus set was informative and could be useful for epidemiological studies of Beijing genotype strains.^50^

However, accounting for strain polymorphism, geographical differences in isolate distribution and variations in the prevalence of Beijing strains in China, the allelic diversity (*h*) of a certain locus varies, with these loci potentially exhibiting different discriminatory powers.^19^ Of the standard 24-locus set, MIRU20, Mtub34, MIRU02 and MIRU24 were excluded from the present study because of their low rates of polymorphism and *h* values <0.1 in Asia.^51^ As reported previously, QUB11a and QUB18, which are also not included in the 24-standard loci, display moderate discriminatory power in Heilongjiang Province and are informative and optimal for *M. tuberculosis* genotyping in this area.^19^ Because it is accepted that loci with either extremely high (*h*>0.8) or low (*h*<0.1) allelic diversity are unsuitable for genotyping because of their variation or low discriminatory power, the three loci exhibiting extremely high allelic diversity (VNTR3820, QUB3232 and VNTR4120) in *M. tuberculosis* isolates from the region should also be omitted. In this study, even within a single region (that is, Heilongjiang Province), we found that the allelic diversity at a particular locus differed between Beijing and non-Beijing strains. Therefore, we analyzed the remaining loci to identify the VNTR locus or combination of loci that was capable of discriminating Beijing- from non-Beijing genotype strains.

The combination of MIRU26, MIRU31 and ETRA was established for the differentiation of Beijing-lineage isolates, with a sensitivity of 94.7% and a specificity of 98.5%.^52^ A previous study showed that Mtub30 and Mtub02 can serve as alternatives for identification of Beijing genotype stains in resource-limited regions.^53^ Compared with genetically divergent non-W-Beijing strains, Beijing genotype strains contained a significantly higher number of tandem repeats in the loci (ETRA, ETR-E, QUB26, QUB18, QUB11B and Mtub21). Furthermore, W-Beijing genomes frequently possess seven, eight or nine tandem repeats at loci QUB26 and QUB18, indicating a pattern that is different from other genotypes.^54^

The above-mentioned studies focused on difference in the number of repeats among all strains at a given locus and their contribution to the differentiation of the Beijing strains. The proportions of each Beijing sublineage categorized according to the repeat number at a particular VNTR locus were applied for the selection of phylogenetically informative VNTR loci based on the dominant percentages of genotypes that exhibited different repeat-number patterns.^40, 55, 56^ This method is based on the repeat-number differences and is less quantitative, whereas another method for choosing a locus combination is based on the highest cumulative HGDI accompanied by successive addition of each MIRU–VNTR locus according to the allele diversity.^57^ As first-line VNTR locus sets for genotyping *M. tuberculosis*, loci with extremely high and low allele diversity should be excluded;^36^ however, some alternative loci might also exhibit the same allele diversity, resulting in difficulties in choosing suitable loci. Furthermore, considering the discriminatory redundancy between loci, we need to try many combinations to obtain the optimal set and the discriminatory power for genotyping *M. tuberculosis* genotypes may be neglected.^36^ In this study, we adopted a new method that considers the APD of a VNTR locus to distinguish the Beijing from non-Beijing strains. This approach is based on the proportional difference between strains having the same repeat number at a certain VNTR locus between Beijing and non-Beijing strains and the accumulative difference of all the repeat patterns. In this method, a locus set is selected according to the APD values in descending order, facilitating the selection of better locus sets for discriminating Beijing and non-Beijing strains.

To classify Beijing sublineages based on the VNTR loci, the proportions of each Beijing sublineage defined by SNPs were analyzed based on the number of repeats at a certain VNTR locus. Ancient and modern Beijing sublineages can be classified by different VNTR loci from different regions (for example, MIRU31 and Mtub04 in Taiwan; Mtub21 and QUB4156 in Japan, Thailand and Peru).^40, 55, 56, 58^ Furthermore, STs of Beijing sublineages defined by SNPs can be distinguished based on the repeat number at a VNTR locus. For example, MIRU10, QUB11b and VNTR3155 display unique repeat numbers in ST3, STK and ST26 strains, respectively.^58^

In our previous study, we found that locally prevalent *M. tuberculosis* strains were modern STs, including ST22 and ST10,^27^ and that some sublineages are genetically close to either ST22 or ST10, whereas ancient sublineages are less dominant. Therefore, in this study, we divided all sublineages into three groups: ST22s, ST10s and ST others (ancient sublineages). Application of APDs to select VNTR loci for the differentiation of Beijing sublineages revealed that this method was efficient for VNTR locus selection. We found that only one locus (QUB11a) was sufficient for differentiating ST10s from ST22s strains, with an APD of 84.3%, whereas the other loci showed lower discriminatory power, with APDs <32%. Five loci (QUB11a, QUB4156, QUB18, Mtub21 and QUB26) were more effective at discriminating ST22s from ST others strains, with APDs ranging from 82.0% to 42.3%. In addition, the five-locus combination showed stronger discriminatory power relative to that observed from using individual loci, and three loci (QUB4156, QUB18 and Mtub21), with APDs ranging from 71.1% to 58.6%, effectively classified strains as ST10s or ST others. Our results suggested that the APD method described here will help to more efficiently select (sub)lineage-discriminating VNTR loci.

In summary, we found that the APD allowed effective determination of the VNTR loci for typing *M. tuberculosis* into different genotypes or sublineages. Possible optimal VNTR locus sets should include VNTR loci with high APD values between the two targeted groups because of the ability of the APD to increase the discriminatory power of the VNTR locus set. The limitation of the present work is that our VNTR loci were selected according to known and previously identified loci. In future work, we will examine additional loci that might exhibit higher discriminatory power and that could be suitable for discriminating *M. tuberculosis* isolates from various areas of the world.

## Figures and Tables

**Table 1:** Allelic diversity (*h*) of different MIRU–VNTR loci

**MIRU–VNTR locus**	**Total (*****n*****=281)**	**Beijing (*****n*****=252)**	**Non-Beijing (*****n*****=29)**
*VNTR3820*	0.890	0.869	0.670
*QUB3232*	0.880	0.855	0.838
*VNTR4120*	0.851	0.833	0.650
QUB11b	0.751	0.736	0.775
QUB26	0.748	0.716	0.768
MIRU26	0.643	0.585	0.672
***QUB11a***	**0.584**	**0.540**	**0.266**
***QUB18***	**0.524**	**0.430**	**0.645**
MIRU31	0.490	0.378	0.494
**Mtub21**	**0.488**	**0.387**	**0.587**
Mtub04	0.478	0.420	0.532
MIRU39	0.465	0.387	0.361
MIRU10	0.367	0.260	0.313
**MIRU40**	**0.338**	**0.274**	**0.640**
Mtub30	0.318	0.195	0.361
**MIRU16**	**0.313**	**0.272**	**0.544**
**MIRU04**	**0.286**	**0.236**	**0.549**
**ETRA**	**0.283**	**0.194**	**0.542**
**Mtub39**	**0.250**	**0.182**	**0.605**
QUB1895	0.178	0.169	0.208
*QUB4156*	0.138	0.132	0.148
*Mtub24*	0.132	0.125	0.145

The italicized text represents the seven newly added loci described in this study. Bold items represent loci exhibiting disparities in *h* values ≥0.2 between the Beijing and non-Beijing family strains.
