# Supplementary material for: A quantitative and efficient approach to select MIRU–VNTR loci based on accumulation of the percentage differences of strains for discriminating divergent Mycobacterium tuberculosis sublineages
Source: Emerg Microbes Infect. 2017 Jul 26;6(7):e68–. doi: 10.1038/emi.2017.58 (PMC5567172; doi:10.1038/emi.2017.58)
Supplement: Supplementary Table S2 [file emi201758x1.docx]

**Supplementary Table S2 Composition of the sequence types in three groups**

| Groups (n) | ST type (No. of strains) | Sublineage |
| --- | --- | --- |
| ST10s (173) | ST10 (159) | Modern |
|  | STF (4) | Modern |
|  | STCH1 (10) | Modern |
| ST22s (61) | ST22 (60) | Modern |
|  | ST8 (1) | Modern |
| ST others (18) | ST25 (3) | Ancient |
|  | ST19 (4) | Ancient |
|  | ST11 (2) | Ancient |
|  | ST26 (5) | Ancient |
|  | ST3 (4) | Ancient |
