# Supplementary material for: A quantitative and efficient approach to select MIRU–VNTR loci based on accumulation of the percentage differences of strains for discriminating divergent Mycobacterium tuberculosis sublineages
Source: Emerg Microbes Infect. 2017 Jul 26;6(7):e68–. doi: 10.1038/emi.2017.58 (PMC5567172; doi:10.1038/emi.2017.58)
Supplement: Supplementary Table S1 [file emi201758x2.pdf]

Supplementary Table S1 The primers in determination of genotypes and sublineages of *Mycobacterium tuberculosis* strains.

Molecular determination	Locus	Nucleotide sequence
genotypes	RD711[a]	Forward: GGCCGCCCTGCTCAAGAACCT
		Reverse: CCTAGGCCGGCGACGAAGTG
	RD750 [a]	Forward: GTCGGCGGTCTGCTTCGTTCC
		Reverse: CCTGTCGGCCGGGTGTCTTTC
	RD105 [b]	Forward: ACAGCGCGGGTCATATCAC
		Reverse: AACCAGCTCCTCGACGCTATC
	RD239 [a]	Forward: GGCCAACATCGACCACCTACCC
		Reverse: ATCCTCGCTACCGGCACCTCAT
	TbD1[c]	Forward: ACGCGGCTGGATGGTGCTGGTTG
		Reverse: CGCGGGCAGGGTCGTCGTGA
	pks15/1 [d]	Forward: GTGTCCTCCTTTGGGATCAG
		Reverse: CTGCCCAGGAAACACGAC
	Rv0679c [e]	Forward: CCGGGAACTAGGAATGGTAA
		Reverse: AGCAACCTCGCAATCTGAC
sublineages	797736 [e]	Forward: GACGGCCGAATCTGACACTG
		Reverse: CCATTCCGGGTGGTCACTG
	909166 [e]	Forward: CGTCGAGCTCCCACTTCTTG
		Reverse: TCGTCGAAGTGGACGAGGAC
	1477596 [e]	Forward: GTCGACAGCGCCAGAAAATG
		Reverse: GCTCCTATGCCACCCAGCAC
	1692069 [e]	Forward: GATTGGCAACTGGCAACAGG
		Reverse: TGGCCGTTTCAGATAGCACAC
	1892017 [e]	Forward: GCTGCACATCATGGGTTGG
		Reverse: GTATCGAGGCCGACGAAAGG
	2376135 [e]	Forward: TCTTGCGACCCGATGTGAAC
		Reverse: GAGCGCAACATGGGTGAGTC
	2532616 [e]	Forward: CCCTTTTCTGCTCGGACACG
		Reverse: GATCGACCTTCGTGCACTGG
	2825581 [e]	Forward: CCTTGGAGCGCAACAAGATG
		Reverse: CTGGCCGGACGATTTTGAAG
	4137829 [e]	Forward: CGTCGCTGCAATTGTCTGG
		Reverse: GGACGCAGTCGCAACAGTTC

[a]Gagneux S, DeRiemer K, Van T, *et al.* Variable host-pathogen compatibility in Mycobacterium tuberculosis. *Proc Natl Acad Sci U S A* 2006; **103:** 2869-2873.

[b]Hanekom M, van der Spuy GD, Streicher E, *et al.* A recently evolved sublineage of the Mycobacterium tuberculosis Beijing strain family is associated with an increased ability to spread and cause disease. *J Clin Microbiol* 2007; **45:** 1483-1490.

[c]Sales ML, Fonseca AA, Jr., Sales EB, *et al.* Evaluation of molecular markers for the diagnosis of Mycobacterium bovis. *Folia Microbiol (Praha)* 2014; **59:** 433-438.

[d]Zenteno-Cuevas R, Silva-Hernandez FX, Mendoza-Damian F, *et al.* Characterisation of pks15/1 in clinical isolates

of Mycobacterium tuberculosis from Mexico. *Mem Inst Oswaldo Cruz* 2013; **108:** 718-723.
[e]Nakajima C, Tamaru A, Rahim Z, *et al.* Simple multiplex PCR assay for identification of Beijing family Mycobacterium tuberculosis isolates with a lineage-specific mutation in Rv0679c. *J Clin Microbiol* 2013; **51:** 2025-2032.
