# Supplementary material for: A quantitative and efficient approach to select MIRU–VNTR loci based on accumulation of the percentage differences of strains for discriminating divergent Mycobacterium tuberculosis sublineages
Source: Emerg Microbes Infect. 2017 Jul 26;6(7):e68–. doi: 10.1038/emi.2017.58 (PMC5567172; doi:10.1038/emi.2017.58)
Supplement: Supplementary Figure S1 [file emi201758x3.pdf]

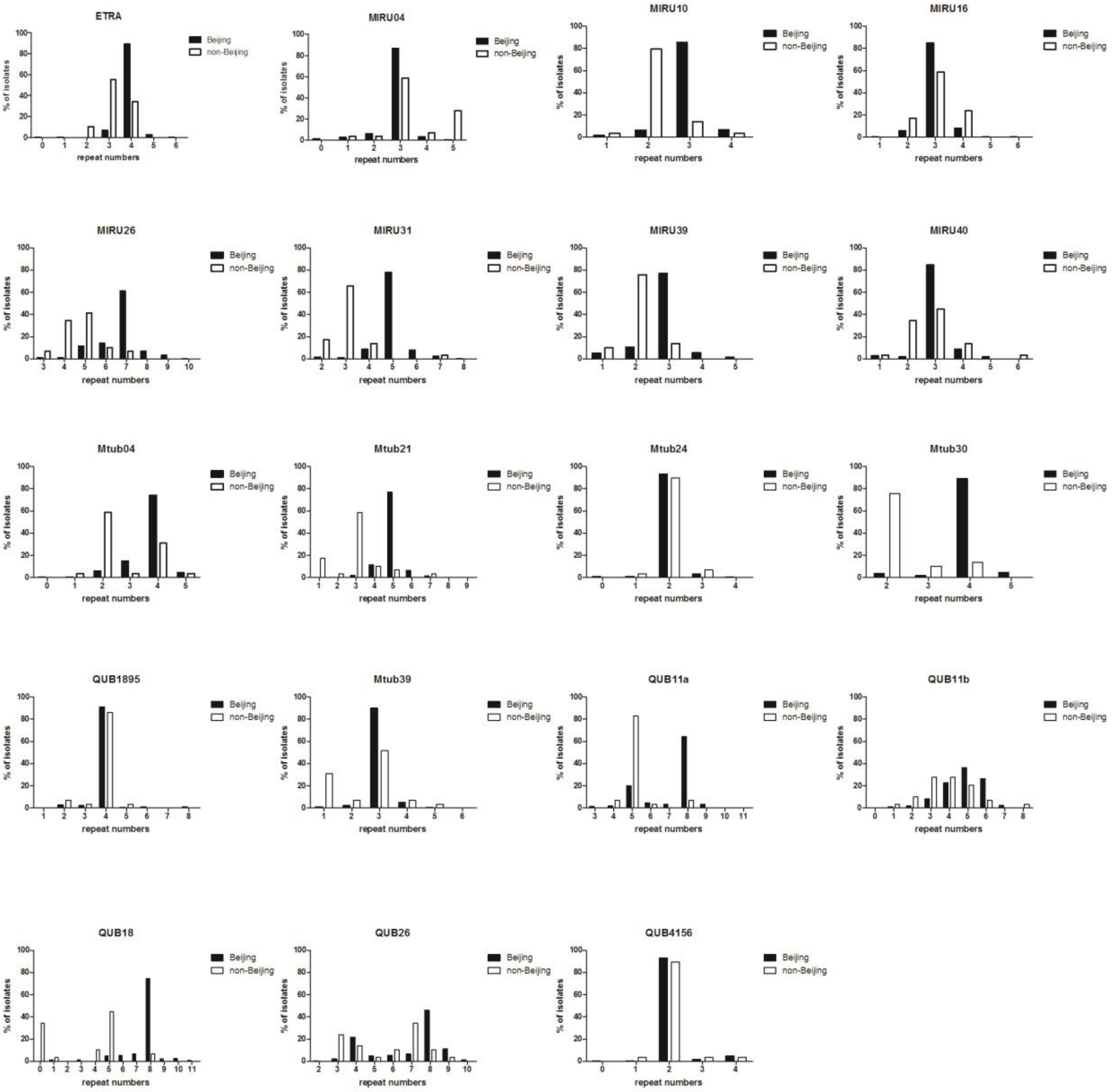

Supplementary Figure S1 The repeat number of 19 VNTR loci respectively between Beijing (black) and non-Beijing strains (white).
